# Six Drivers to Face the XXI Century Challenges and Build the New Healthcare System: “La Salute in Movimento” Manifesto

**DOI:** 10.3389/fpubh.2022.876625

**Published:** 2022-06-29

**Authors:** Francesco Blasi, Enrico Gianluca Caiani, Matteo Giuseppe Cereda, Daniela Donetti, Marco Montorsi, Vincenzo Panella, Gaia Panina, Felicia Pelagalli, Elisabetta Speroni

**Affiliations:** ^1^Department of Pathophysiology and Transplantation, Università degli Studi di Milano, Milan, Italy; ^2^Internal Medicine Department and Respiratory Unit, Fondazione IRCCS Cà Granda Ospedale Maggiore Policlinico di Milano, Milan, Italy; ^3^Department of Electronics, Information and Biomedical Engineering Department, Politecnico di Milano, Milan, Italy; ^4^Eye Clinic, ASST Fatebenefratelli Sacco, Milan, Italy; ^5^Azienda Sanitaria Locale di Viterbo, Viterbo, Italy; ^6^Department of Biomedical Sciences, Humanitas University, Pieve Emanuele, Italy; ^7^Department of Surgery, Humanitas Research Hospital IRCCS, Milan, Italy; ^8^Order of Malta Healthcare Network – Rome, Rome, Italy; ^9^Novartis Farma SpA, Origgio, Italy; ^10^Culture srl, Rome, Italy; ^11^Sapienza University, Rome, Italy

**Keywords:** data analysis, digital technology, health, healthcare system, science literacy, COVID-19

## Abstract

The aging of the population, the burden of chronic diseases, possible new pandemics are among the challenges for healthcare in the XXI century. To face them, technological innovations and the national recovery and resilience plan within the European Union can represent opportunities to implement changes and renovate the current healthcare system in Italy, in an effort to guarantee equal access to health services. Considering such scenario, a panel of Italian experts gathered in a multidisciplinary Think Tank to discuss possible design of concepts at the basis of a new healthcare system. These ideas were summarized in a manifesto with six drivers for change: vision, governance, competence, intelligence, humanity and relationship. Each driver was linked to an action to actively move toward a new healthcare system based on trust between science, citizens and institutions.

## Introduction

Health is a central concept for human beings, and good health is both a necessity and a right. The World Health Organization defines health as “a state of complete physical, mental, and social well-being not merely the absence of disease or infirmity” (1). Accordingly, health includes both the prevention of diseases and maintenance of well-being in every aspect of life, from physical to mental health and the ability to participate in social activities.

The XXI century has already posed important challenges for the management of health. The COVID-19 pandemic highlighted the strong interconnections between health, policy decision making and professional and social activities, underlining critical points of the current European and Italian healthcare systems. Indeed, COVID-19 has stressed healthcare system globally, urging a series of interventions spanning from economic policies to governance and ethics, implying collaborations between countries and among different institutions within the same country (2, 3). This unprecedented event added additional challenges for the healthcare system, such as the stable increase of the world's population (4) and its aging (5) together with the growing burden of chronic diseases (6).

The development of a new, composite healthcare system represents the path for health management in the new century with the need for programs that facilitate the approach to the dimension of health (7, 8). In addition, the pandemic has shown how new digital solutions (telemedicine, wearables, artificial intelligence) can help in improving the quality of health and well-being. These new technologies have provided strategic support for healthcare systems to reach a greater proportion of the population, assuring more streamlined monitoring and assistance within both the national context and in a shared European scenario (9, 10). The recent finalization of national recovery and resilience plans within the European Union (11, 12) is an unprecedented opportunity for development and reforms for Italy to implement and renovate its public administration and healthcare system, in the effort to minimize social differences and guarantee equal access to health services.

To provide suggestions aiming to reach these goals with specific reference to the Italian healthcare system, a multidisciplinary Think Tank was set up by several representatives of Italian Institutions, private company, academia and associations within the social project called “La Salute in Movimento”. Starting from the general evaluation of the current Italian situation, and with an eye to the international scenario, the Think Tank explored new ideas and proposals to be implemented in the Italian healthcare system. Herein, we report the statements of the project, which calls for objectives and actions that may be helpful to policymakers to highlight the need for a systemic approach to health issues, to contribute to the generation of a more modern and sustainable healthcare system.

## Policy Options and Implications

### The Six Drivers for Change: The Pillars for a New Concept of Health

A panel of experts and key opinion leaders belonging to different areas within the Italian society (academia, medicine, pharmaceutical industry, philosophy, psychology, technology, non-profit organizations) gathered in a multidisciplinary Think Tank to discuss ideas and critical points to face the new challenges of the XXI century, and to design a vision for a renewed Italian healthcare system. This discussion started from considerations on the building blocks of the health system as described by the WHO (service delivery, health workforce, health information systems, access to essential medicines, financing, and leadership/governance) (13) and followed the related issues pointed out by each discussant.

The panelists organized their discussion into a manifesto summarizing the features of global, sustainable, inclusive healthcare in six actions, named the drivers for change, to improve the Italian healthcare system and to propel the transition toward it: vision, governance, competence, intelligence, humanity and relationship.

Artificial intelligence (AI) and data analysis are tools crossing the different fields, and identified as strategic to create opportunities for the challenges toward concrete actions.

### Vision

The vision for the future of the healthcare system considers health as a dynamic process, which is connected to science, social relationships, education and technology. The link between health and science must be re-discovered by the general public. This new perception will likely contribute to prevent mistrust about new discoveries, resulting in practical advantages to the healthcare system and society. To reach this goal, it is crucial to promote a global health literacy alliance, bolstering the ability to access, understand and make use of scientific information by the population (8, 14, 15). Without the ability to understand the benefits provided by treatment, even the most advanced therapies fail in providing care, because they are likely ignored or avoided. Indeed, several lines of evidence have demonstrated the connection between low health literacy and overall low utilization of healthcare solutions, thus resulting in worse health outcomes for the population (16). This is particularly true in Italy, and recent studies highlighted the overall inadequate health literacy of the population compared to other European countries (17, 18). This situation exacerbated during the pandemic as demonstrated by the limited literacy concerning, for instance, vaccines and their potential benefit for the society (19).

The scientific method, being experimental, transparent and repeatable, is a solid means of producing the knowledge which is at the basis of our societies. The clashes about the COVID-19 response have revealed that the scientific method is largely ignored, and that this ignorance has in-depth, heavy social effects. Therefore, we need to popularize the scientific method (20). Spreading knowledge about the scientific method to the public requires an efficient communication plan that involves the educational system; only through diffuse intervention can the general public possess the tools needed to augment trust toward the scientific process and its applications to healthcare. This will lead to informed participation about health-related issues, allowing to better overcome the challenges of both communicable and non-communicable diseases. A critical part of this process consists in the definition of effective and reliable tools to monitor and measure health literacy, and identify the critical factors that may interfere with the implementation of the process (e.g., ability to discern correct information, reverse the lack of confidence of individuals in using information) (21).

### Governance

An efficient health system is based on efficient governance that ensures the development of strategic plans for health assistance. The improvement of health policies has been faced by European countries and can be reached by an appropriate governance (22). One of the main issues for the Italian healthcare assistance is a plan for the territorial primary care providing patients with the possibility to be assisted at home. There is the urgent need to better integrate the activities of local general practitioners with hospitals to ensure adequate assistance, especially in case of health emergencies and chronic disease. A territorial health service, managed with the support of telemedicine and remote patient monitoring, will likely allow equal access to therapies and increase adherence to treatment. The efficiency of telemedicine has been reported after COVID-19 pandemic in several countries, and a similar effect is foreseen also for the Italian scenario (23). The strengthening of home care and territorial organization of the healthcare service is one of the indications included in the Italian National Recovery and Resilience Plan (NRRP) (12, 24).

### Competence

Digital technology is a powerful tool to enhance the quality and efficiency of healthcare and the WHO recommended the use of digital interventions for the implementation of health systems (25, 26). Nevertheless, the ability of the healthcare system and healthcare professionals (HCPs) in adopting digital health solutions to implement services and patient assistance has been reported to be slow in US, Europe and Australia (27–29). Studies analyzing the main gaps that prevent the embracement of digital health have highlighted the need of appropriate and up-to-date competencies and digital literacy among primary care providers (30, 31). Interestingly, the improvement of basic IT knowledge and skills for HCPs are reported to be central facets (31). Despite the great advances undertaken upon the pandemic, the young generation of Italian physicians is still in need of an adequate education and training with respect to digital competence (32).

To take advantage of scientific and technological innovations, the Italian national health system should invest, considering the directions of NRRP and collaboration with private partners, in a wide digital education training plan directed to all HCPs, aiming to optimize their contribution to this new model of healthcare.

The need for health literacy should also be considered for stakeholders such as policy makers as a competence shared within all sectors involved in the healthcare system that results in generalized effective improvement and equity of the system (33). The development of a technological ecosystem integrated within national institutions that can be used for digital education has been already translated into practice in some realities, as it happened in Brazil following the pandemic. In that case, among other interventions, an online platform shared among the stakeholders and the institutions was useful to organize and capillary spread the right information about the pandemic. The technological ecosystem allowed the education and update of local HCPs, reducing the potential differences in the access to information due to different geographical areas, which would introduce disparities in case of face-to-face education (34).

Universities play a crucial role in education of new generations in fostering human centered innovation, adopting open science policy and strengthening civic engagement (35), as well as adapting their offer to cope with the new needs in the healthcare. In Italy, new courses based on multidisciplinary curricula (e.g., medical and technical schools, combining medicine and engineering) could educate the new HCPs of the future. The aim is also to enable systematic and easier creation of multidisciplinary medical teams like those that were forcedly improvised during COVID-19 and which will be required by the more sophisticated treatments that the future will bring.

### Intelligence

AI and machine learning are believed to become essential components of medical research and improve healthcare efficiency (36). Nevertheless, current evaluation of the impact of AI and machine and deep learning in clinical practice reveals the limitations of such algorithms. The use of machine learning is mainly done in retrospective studies, and both the type of input data and the lack of transparency by which the output is generated are currently a major drawback in the broad application of this technique (37). To overcome these limitations, human intelligence should walk side by side with AI to deal with the possible bias generated by machine algorithms, without delegating decisions to them (38). Telemedicine and digital therapies rapidly spread in daily health management with the COVID-19 emergency and are here to stay (23). New advanced digital tools provide HCPs with an unprecedented amount of data that needs to be safely collected and analyzed for real-world evaluations, without forgetting ethical aspects (39, 40). This data represents a valuable driver of innovation in medicine and healthcare when they are rigorously collected and used according to appropriate methodology and ethical aspects (41). The presence of a government body for the technical and operative support of healthcare policies (national agency for regional health services, AGENAS) is a peculiarity of the Italian system and should be better exploited as a reference for data collection and analysis. This would contribute to assure reliable and high-quality health outcomes.

### Humanity

Health is an inclusive concept. The new health system should take advantages from new technologies without forgetting human values, social justice and the environmental impact (33). Healthcare services should be delivered through improved cooperation of both healthcare and social services and be inclusive (42). COVID-19 highlighted the value of humanity, collaboration and inclusion for global well-being, with the necessity to build an accessible healthcare system that guarantees the best treatment for everyone (43). One of the aspects seen during the COVID-19 pandemic is the value of caring patients at home. Remote home monitoring and care for different pathologies increased during the pandemic also in Italy (44, 45), but there is still room for amelioration of the service. Indeed, a review analyzing the experience of remote home care revealed how the models proposed lack standardization and acquisition of proper data, and need a strong and inclusive patient engagement to become effective (46).

### Relationship

An efficient healthcare system relates to scientific institutions and governments (22). The positive interaction between the patient and all the physicians involved in the care process is at the basis for proficient management of any condition, as well as the trustworthy cooperation between HCPs and policy makers, and the connection and strong relationship between local care activities and the hospital system (47). The establishment of functional relationships between all stakeholders should influence the definition of best practice and therapeutic paths with the involvement of patient associations and caregivers (48–50). The health system should be evaluated according to the feedback from patients and the improvements that such indications can provide to HCPs and the general service offered (51).

### Call to Action

How should a new global, human health system be designed? The panel of experts defined some actions to be taken to reach this bold objective. These actions are in accordance to the NextGenerationEU project (24) and have constituted the subject of a broader debate in a virtual 2-day event, Agorà, with stakeholders, healthcare and academia professionals, policy makers, patient representatives and the general public.^1^ Within each of the manifesto's six overarching principles, the discussants worked in breakout sessions to define these actions. The use of the new technologies represented a common factor, given that innovation is central in the development of a new concept of health.

The final outcomes of each of the initial six principles are presented below.

#### Vision: Develop a Modern Scientific Communication Model

The Faro Convention encourages “citizens to recognize the importance of cultural heritage objects and sites through the meanings and values that these elements represent for them” (52). The broader and contemporary perspective we are promoting requires that these objects and sites, as well as cultural practices and values, include not only those of art but also those of science, since they contribute to both individual and social well-being. Thus, the concept of cultural heritage should be extended to medications and scientific innovation to underline their central role in the development of a culture of wellness.

The dissemination of health concepts, science and scientific research needs to be facilitated by the use of a new format based on the current popular language, from TV series to videogames, with the aim of reaching the widest possible audience. New accessibility to science will re-shape the public's perception, making it clear that scientific progress is reached based on evidence that is collected through a trial-and-error path.

A new perception of science will stimulate reciprocal empathy and trust of citizens toward HCPs and scientists, encouraging citizens to actively participate in the management of their health.

#### Governance: Build a New System for the Challenges of the Future

Establish a new Scientific and Technological Impact Assessment Body, an institutional organization to support policy makers in both legislative and executive functions. Indeed, the massive scientific and technological changes in the healthcare scenario urge for the need of scientific support for decision makers. A capable and dedicated scientific institutional body is also needed for the proficient management of funds from the national recovery and resilience plan, as it is necessary to have broad knowledge of the scientific scenario to sustain informed solutions.

#### Competence: Promote Health Culture as a Source for Solutions to Complex Issues

Creation of a new platform, through collaboration with scientific faculties of universities that is accessible to HCPs for both working and training. This platform, which we suggest can be called Formative Ecosystem for Healthcare Innovation (FEHI), will allow a constant update of the requested competencies for HCPs (31), delivering services such as the acquisition of certifications, both online (providing educational activities through Universities, IRCSS/Centers of Excellence, private societies), and in physical presence (building of a network of centers that will assist in the organization of educational activities). The platform will bring the available competencies to the attention of policy makers and the national healthcare system to match professionals with the correct job function. The new professionals should be trained to better understand and use new technologies, data science, AI and behavioral change models. The need for a more comprehensive inclusion of digital health-related topics has been also highlighted by the European Medical Students Association and described as a result of a recent study (53).

#### Intelligence: Human and Artificial Intelligence Must Cooperate in Data Management

Obtaining wide access to healthcare data from the entire country, collecting it from both public and private structures through the different 21 regional electronic health record systems (“Fascicolo Elettronico Regionale”) (54) can be a new mission for the national healthcare system. Data organized in a centralized national system that is accessible to all healthcare centers will allow its utilization for its primary use (i.e., make it available to the patient and physicians when needed) as well as for secondary uses (i.e., research).

Moreover, the healthcare system should promote the creation of a network of specialists, general practitioners, patients and med-tech companies that shares valuable data for public health. Neglected use of the available data may result in a loss of efficacy for the healthcare system, with a waste of both resources and opportunities (55), even if any access to private information should take place according to ethical principles and current EU privacy regulations (56).

Lastly, the use of AI should be considered as a tool to increase the scale of care, through the identification of models or algorithms on which to base patient care in the daily practice in order to provide the same access to treatment to every patient, thus reducing inequities (57).

#### Humanity: Building a Healthcare System That Is Closer to the Patient

An integrated home assistance service for non-self-sufficient elderly, children and frail subjects should be favored. This should go beyond the idea of the hospital as the only feasible place of care, thanks to the help of new technologies and the development of digital platforms to provide room for storytelling (24). This objective is central in the NRRP (12), which clearly states the need to create conditions that will allow the patient to be cured at home, strengthening home assistance and use of telemedicine. In this light, patients and pathologies suitable for home treatment have to be defined, together with the identification of parameters that can be remotely monitored and alerting systems that allow remote interaction between patients, caregivers and HCPs.

#### Relationship: Overcoming Individual Visions in the Healthcare System

A “logbook” of the patient to establish a network of connections within the healthcare system should be implemented. This network will easily allow for constant updates with the healthcare activities of each patient, and will provide indications on the type of procedure needed or performed, the HCP in charge of the procedure and the outcome. The new “logbook” function will integrate the personal electronic health record that is already in use within the different regional healthcare systems.

## Conclusions

Herein, we have outlined proposals to build a renewed model of healthcare for Italy that is deeply inspired by two basic principles that characterized the project “La Salute in Movimento”: relationship, with its scope of collaboration and sharing, and trust, as an essential step to achieve innovation. These principles need to be structurally implemented on three fronts: data governance, status of algorithms and digital skills. Notably, technical and digital improvements are actually needed to humanize healthcare. Besides the limitations of the current study, which was carried on without following a structured methodological approach, the participation of experts in different areas involved in the development, support and maintenance of healthcare solutions and the integration of the diverse perspectives made the presented points of value in the current policy debate for the improvement of health care in Italy, and could serve as inspirational also for other similar efforts in different countries.

We aim to contribute to the implementation of the six actions of the manifesto to build the new system:

1. The infosphere consists of a network of “intelligent” nodes. The era of closed and self-referential contexts is no longer viable. Debate is public and takes place on different platforms.
2. Open debate can foster communication among scientists, and between the scientific community and the public (Open Science).
3. Communication matters. Scientists do not always know how to communicate.
4. It is important to spread the culture of science (health literacy and science literacy).
5. It is important to understand the scientific method, its complexity and foster critical thinking.
6. There is the need for education aimed at social platforms, places of communication and participation.

## Author Contributions

FB, EC, MC, DD, MM, VP, GP, FP, and ES conceptualized, wrote the original draft, reviewed, edited, and approved the final manuscript. All authors contributed to the article and approved the submitted version.

## Funding

Novartis Farma Italy organized Think Tank and provided support for the preparation of this article, but did not pay any honoraria to the authors. Editorial support was provided by Barbara Bartolini, Ph.D., and Patrick Moore of Health Publishing and Services Srl.

## Conflict of Interest

FB reports grants and personal fees from AstraZeneca, Bayer, Chiesi, GSK, Menarini and Pfizer, personal fees from Grifols, Guidotti, Insmed, Novartis, Zambon, Vertex and Viatris. EC reports personal fees from Servier, Novartis, MSD and Medtronic. MC reports personal fees from Novartis, Bayer and Alcon and to be co-founder of MgShell srl. MM reports personal fees from Baxter and MSD. GP and ES are employees of Novartis Farma. The remaining authors declare that the research was conducted in the absence of any commercial or financial relationships that could be construed as a potential conflict of interest.

## Publisher's Note

All claims expressed in this article are solely those of the authors and do not necessarily represent those of their affiliated organizations, or those of the publisher, the editors and the reviewers. Any product that may be evaluated in this article, or claim that may be made by its manufacturer, is not guaranteed or endorsed by the publisher.

## Footnotes

^1^ https://lasaluteinmovimento.it/
